# A novel approach to phylogenetic tree construction using stochastic optimization and clustering

**DOI:** 10.1186/1471-2105-7-S4-S24

**Published:** 2006-12-12

**Authors:** Ling Qin, Yixin Chen, Yi Pan, Ling Chen

**Affiliations:** 1 Department of Computer Science, Nanjing University of Aeronautics and Astronautics, Nanjing, 210096, China; 2 Department of Computer Science and Engineering, Washington University in St.Louis, USA; 3 Department of Computer Science, Georgia State University,34 Peachtree Street, Suite 1450, Atlanta, GA 30302-4110, USA; 4 Department of Computer Science, Yangzhou University, Yangzhou, 225009 China

## Abstract

**Background:**

The problem of inferring the evolutionary history and constructing the phylogenetic tree with high performance has become one of the major problems in computational biology.

**Results:**

A new phylogenetic tree construction method from a given set of objects (proteins, species, etc.) is presented. As an extension of ant colony optimization, this method proposes an adaptive phylogenetic clustering algorithm based on a digraph to find a tree structure that defines the ancestral relationships among the given objects.

**Conclusion:**

Our phylogenetic tree construction method is tested to compare its results with that of the genetic algorithm (GA). Experimental results show that our algorithm converges much faster and also achieves higher quality than GA.

## Background

An evolutionary tree, or phylogenetic tree, is a model of the evolutionary history for a set of species. With more and more DNA and protein sequences have been obtained [1-3], the problem of inferring the evolutionary history and constructing the phylogenetic tree has become one of the major problems in computational biology. This is because the evolutionary relationship of species provides a great deal of information about their biochemical machinery. For example, RNA's secondary structure is most accurately determined by selecting correlated mutations of a class of related species.

A phylogenetic tree is a tree showing the evolutionary interrelationships among various species or other entities that are believed to have a common ancestor. In a phylogenetic tree, each node with descendants represents the most recent common ancestor of the descendants, and the edge lengths correspond to time estimates. Each node in a phylogenetic tree is called a taxonomic unit, and the leaves usually denote a set of objects (proteins, species, etc.). Internal nodes are generally referred to as Hypothetical Taxonomic Units (HTUs) as they cannot be directly observed [3-6].

To construct a tree from a set of species, one must have a metric to decide if a tree is better than another one. Many criteria have been proposed. But in general, they turn out to be NP-hard to optimize. There is still no consensus in the biology community on how to make a good tree.

One way to counteract this problem is to execute many different phylogenetic clustering methods resulting in various starting tree topologies. A choice from the generated trees gives rise to the best one. Another way to handle this problem is to use a global optimization technique to derive the optimal topology of the tree. In this paper, ant colony algorithm is applied both as a clustering method and as a global optimization technique so that the optimal tree can be found even with a bad initial tree topology.

During the past years, a number of efforts have been contributed to phylogenetic analyses using genomic sequences, which could be either whole genomes (complete gene sequence sets) or complete protein sequence sets [7-9]. There are three main methods for constructing phylogenetic trees: distance-based methods such as neighbour-joining, parsimony-based methods such as maximum parsimony, and character-based methods such as maximum likelihood or Bayesian inference [1,2]. The distance based approaches avoid the high computational complexity of multiple sequence alignment (including genome reorganization) to compute an evolutionary distance and try to construct the phylogenetic trees efficiently. The phylogenetic clustering method in this article belongs to the distance based category.

Ant Colony Optimization (ACO) is a new evolution simulation algorithm proposed by Italian researchers Dorigo et al [10]. Inspired by studies on biological ant colony, they recognize the similarities between the ants' food-hunting activities and TSP, and successfully resolve the TSP problems using the same principle that the ants have used to find the shortest route to food source via communication and cooperation, and it has been applied to lots of combinational optimization problems [10,11].

**Figure 1:**
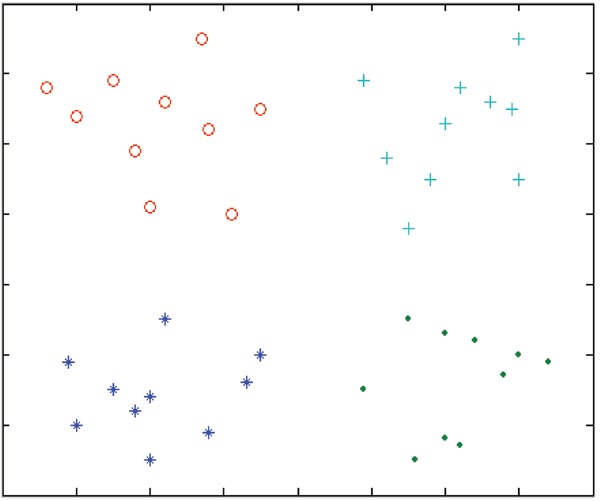
**The initial objects**. To illustrate the novel clustering process by artificial ants, we test a data set with four data types each of which consists of 10 two-dimensional object (x, y) which belong to four classes as shown in Fig. 1. Here x and y obey normal distribution *N*(*u*, σ ^2^). The normal distributions of the four types of data (x, y) are [N(0.2,0.12), N(0.2,0.12)], [N(0.6,0.12), N(0.2,0.12)], [N(0.2,0.12), N(0.6,0.12)], [N(0.6,0.12), N(0.6,0.12)] respectively.

**Figure 2:**
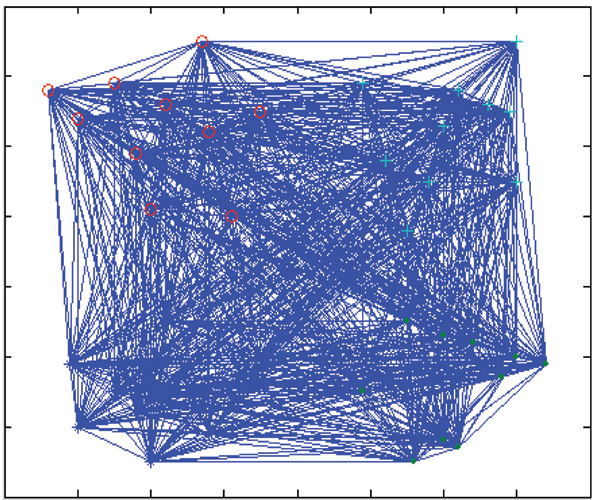
**The initial pheromone digraph**. Fig. 2 shows the initial pheromone digraph of this object set. The pheromone digraph obtained after 50 iterations of AAPTC is then modified by omitting the edges whose pheromone value is less than ε = 1.95.

**Figure 3:**
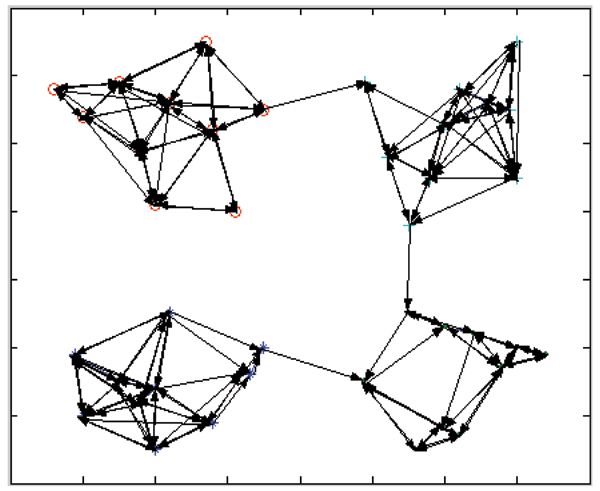
**The modified pheromone digraph (after 50iterations)**. The modified digraph is shown in Fig. 3, objects with lower similarity form some strong connected components

**Figure 4:**
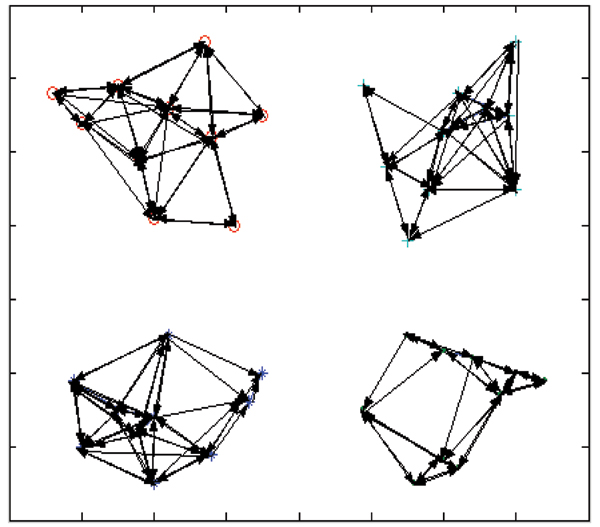
**The finial strong connected components**. As shown in Fig. 4, four strong connected components of the modified digraph are computed which form the finial clusters.

We note that in the ant colony algorithm, ants can volatilize a kind of chemical odour called pheromone when they encounter each other or in the process of seeking their fellows. Enlightened by this fact, we first apply weights of rejection and acceptance between the objects to form a complete digraph in which the vertexes represent the objects and the initial weight of each edge between vertexes is the weights of acceptance between the objects. The novel clustering process by artificial ants is illustrated in Fig. 1, 2, 3 and 4, during the process, the pheromone on each edge of the digraph will be updated with the artificial ants' adaptive movements, and some adaptive strategies are also presented to speed up the clustering progress. Finally the clusters got by the ants are used to progressively construct the phylogenetic trees.

## Results

### Constructing a specific digraph for the objects

Ants can volatilize a kind of chemical odour called pheromone when they encounter each other or in the process of seeking their fellows. Based on this kind of odour, ants will naturally attract those who have similar features and repel those that are different. In this paper, artificial ants were set to travel on the graph and deposits pheromone on the edges they passed. As showed in Fig. 1 and Fig. 2, in each step, the artificial ant selects the next vertex according to the acceptance weight in digraph and some heuristic information. The pheromone on each edge of the digraph will be updated with the artificial ants' adaptive movements, and some adaptive strategies are also presented to speed up the clustering progress.

### Strong component analysis

The more similar the objects are, the higher the quantity of pheromone may be deposited on the edge between their vertexes. To make full use of the quantity of pheromone on each edge, we omit some connections whose pheromone value is less than a certain threshold to get a new digraph, and the strong connected components of the new digraph forms the finial clusters. This way, the initial objects are separated into a few clusters by the ant sub-colony. Finally these clusters obtained by the ants are used to construct the phylogenetic trees progressively.

### Optimizing the phelogenetic trees

Artificial ants in the same sub-colony try to construct an independent phylogenetic tree as a solution of the problem by their cooperation; and different sub-colonies construct different trees so as to maintain diversity of candidates. After optimizing these trees, the performance of these solutions is improved. Meanwhile, the pheromones on the edges of high fitness valued trees are increased to strengthen the ants' clustering process.

The phylogenetic tree construction method showed in this paper is tested to compare its results with that of GA, experimental results show that our algorithm is easier to implement and more efficient. Comparing to GA, it can converge much faster and obtain higher solution quality.

## Discussion

Reconstruction of the phylogeny is one of the most important problems in evolutionary study, which is very difficult for large data sets in macromolecular databases. The number of possible phylogenetic trees is exponentially large and the space of topologies cannot be searched exhaustively. Even heuristic searches can be very slow in this case, especially when computationally intensive optimality criteria such as maximum likelihood (ML) are used.

An exhaustive search for the ML topology is usually computationally prohibitive for more than 20 taxa (species). At the same time, the clustering approach for a phylogeny inference is advantageous for a number of reasons, including the ability to model a variety of factors affecting nucleotide sequence evolution, robustness to violations of model assumptions, and resistance to long branch attractions. The use of stochastic algorithm provides an opportunity to develop new efficient and fast methods for phylogeny analysis.

## Conclusion

The proposed adaptive ant colony algorithm for phylogenetic tree construction method (AAPTC) consists of three components, including initialization, constructing phylogenetic trees through clustering, and phylogenetic tree optimization.

In the stage of initialization, a weighted digraph is built where the vertices represent the data to be clustered and the weight is the acceptance rate between the two objects it connected.

In the course of constructing the phylogenetic trees, the ants travel in the digraph and update the pheromone on the paths it passed. At each step, ants choose the next vertex according to a certain probability depending on the pheromone and the heuristic information of the edge. The digraph is first modified by omitting some edges whose pheromone value is less than a threshold, and then the strong connected components of the updated digraph are computed to form the clusters which are used to construct the phylogenetic trees.

After getting a group of phylogenetic trees, the ant colony and its pheromone feedback system act as a global optimization technique to derive the optimal topology of the phylogenetic tree.

The algorithm showed in this paper is tested using randomly generated sequences. Using the same sequences, we also test the GA method. Our experiments were implemented on Dell Precession workstation 380 with IntelP4 Hyper Threading Processor of 3.2 GHz and 800 M Front Bus Speed.

As showed in [3], the simulated data sets used are generated in two different ways. The first set of simulated data consists of trees where the topology of the tree is fixed and randomly generated branch lengths are assigned to each node-to-node connection. The resulting distance matrix is used as input for the methods to be tested. In this way four sets were generated (S11, S12, S13 and S14) which consists of distance matrices defining ancestral relationships among 24, 96, 1000, and 4000 objects, respectively. The second sets S21, S22, S23 and S24 include stochastically generated distance matrices. For these data sets, the optimal tree is not known.

In fact, AAPTC not only provides a novel clustering method to obtain a group of good initial tree topologies, but also has global optimization on these trees. In this way, the AAPTC produces an ensemble of trees of almost similar quality. Whereas the GA method cannot guarantee the topology quality since its sole initial tree topology was generated by some other clustering methods such as NJ or FITCH. The ensemble of high qualified solutions of AAPTC allows experts to decide which topology is most likely since the quality criterion (the fitness value) used does not guarantee the optimal tree topology.

**Figure 5:**
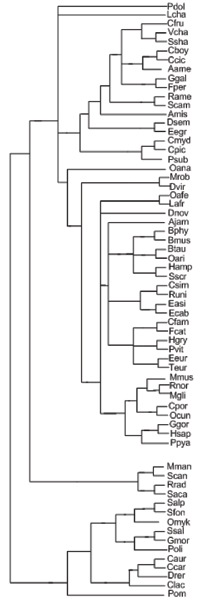
**The ant colony based phylogeny on the 64vertebrates**. Using the vertebrate dataset [8, 16], the ant colony based phylogeny is showed in fig. 5, we can see that the constructed phylogeny is largely consistent with the taxonomy tree showed in[8]. For example, all perissodactyls, birds, reptiles, bony fish etc. are grouped together, as they showed in neighbour join based phylogeny and the taxonomy tree of [8]. This demonstrates that our ant colony based phylogenies at least can equally performance with NJ based method. And from tables of experimental results we can easily find that ant colony based phylogeny has an advantage of easily computed and optimized results.

Tables 1, 2, 3 and 4 show the performance comparison of the two methods. In all the tables, the performance of a method is measured by the fitness value between the original and calculated distance matrices. The number of examined trees is depicted in parentheses. For AAPTC the mean, standard deviation, highest, and lowest fitness value derived from 50 independent trials are given. The basic parameters are set as m = n ρ = 0.05 C = 10 q0 = 0.95. We also use the vertebrate dataset [5,12] to evaluate the performance of our algorithm. Vertebrate database contains in total 832 mitochondrial proteins from 64 vertebrates. The results of the neighbour join based phylogeny and taxonomy tree is shown in [8], and ant colony based phylogeny is shown as fig 5.

## Methods

### Ant Colony Algorithm

Here we briefly introduce AC and its applications using TSP as an example. In the TSP, a given set of n cities has to be visited exactly once and the tour ends in the initial city. We denote the edge between city i and j as (i, j) and its distance as d_ij _(i, j ∈ [1, n]). Let τ_ij_(t) be the intensity of pheromone on (i, j) at time t, and use τ_ij_(t) to simulate the pheromone of real ants. Suppose m is the total number of ants, at time t the kth ant selects from its current city i to city j according to the following probability distribution:



Where allowedk is a set of the cities can be chosen by the kth ant at city i for the next step, η_ij _is a heuristic function which is defined as the visibility of the link between cities i and j, for instance it can defined as 1/*d*_*ij*_.

The relative influence of the trail information τ_ij_(t) and the visibility η_ij _are determined by the parameters α, β. When α = 1 and β = 0, the algorithm becomes a complete heuristic algorithm with positive feedback and when α = 0 and β = 1, it is just a traditional greedy algorithm. For every ant, its path traversing all the cities forms a solution. The intensity of pheromone is updated by Eq. (2):

*τ*_*ij*_(*t *+ 1) = *ρ*τ_*ij*_(*t*)+Δ*τ*_*ij *_    (2)

Where 0<ρ<1 represents the evaporation of τ_ij_(t) between time t and t+1, Δτ_ij _is the increment of the pheromone on (i, j) in step t, and Δτ_ij_^k ^is the pheromone laid by the kth ant on it, it takes different formula depending on the model used.



For example, in the most popularly used model called "ant circle system", it is given as Eq.(4).



where Q is a constant and L_k _is the total length travelled by the k^th ^ant.

### Constructing phylogenetic trees by clustering and optimization

The overall algorithm for constructing the phylogenetic trees is given below.

Begin

1 Initialization

1.1 Initialize parameters: minC, m ε, γ, α, β;

1.2 Initialize the pheromone digraph;

1.3 For each ant in each sub-colony do

Chooses an initial object to visit randomly;

End for

2 While (not termination) do // 500 iterations

2.1 For each sub-colony do //m sub-colonies

2.1.1 Set a root node rt as the ancestor of all the objects;and let CO denote the current object set, the initial value of CO equals the given object set of the problem

2.1.2 While (not termination) do

//500 interations

For each ant k in current sub-colony do

//m ants

While (allowed_k _not empty) do

Compute probability function p;

Select the next object j to visit;

allowedk =allowedk -{ *j *};

End while

Reset allowedk= CO;

End for

Have local pheromone updating on each edge in the digraph according to the evolutionary distance between objects;

Adaptively update the parameters of α, β;

End while

2.1.3 Transfer the pheromone digraph to another digraph by omitting the edges whose pheromone value is less thanε ; find out the strongest connected components of the updated digraph as clusters. Join the small clusters with the nearest cluster till there are two clusters left, we denote them as clu1 and clu2;

2.1.4 let clu1 and clu2 be the internal node to denote the children of the root node rt

2.1.5 If the number of objects in clu1 is lower than 1 let rt=clu1, CO = { clu1} break;

2.1.6 If the number of objects in clu2 is lower than 1 let rt=clu1, CO = { clu2} break;

2.2 obtain each phylogenetic tree constructed by each sub-colony

End for

2.3 Calculate the fitness value of each sub-colony

2.4 Have crossover and mutation operation to improve the quality of the trees

2.5 Have global pheromone updating operation according to the fitness value of the constructed phylogenetic trees

End while

3 Output the phylogenetic trees constructed by the colony

End

In the while loop between line 2 and line 3, based on τ_ave_, the parameter of threshold ε could be defined as ε = γ*τ_ave_, where γ is a constant. The population size of the ant colony m is normally equal to n/2, here n is the number of the given objects. The value of parameters α and β are subject to be adjusted adaptively in process of the algorithm. In line of 2.4, the crossover and mutation operation are executed by branch moving, swapping techniques introduced in [13].

### Initialization of the Pheromone Diagraph

The initialization stage of the algorithm constructs a weighted digraph with the vertexes representing the given objects and the weighted edges between vertexes representing the acceptance weight between the two objects it connected. The acceptance weight between two objects can be calculated from the evolutionary distance between the objects.

Definition 1: The set of objects

A set of n objects is defined as S=(CO, RT) where *CO *= {*object*_1_, *object*_2_,...*object*_*n*_} represents the object set, and rt is the ancestor of all the objects in CO.

A similarity or evolutionary distance is often obtained by pair-wise comparisons of DNA or protein sequences. The measurements of the evolutionary distance can be classified into the following three categories: the first type usually let the number of homologous genes divided by the total number of genes, or its variants be the evolutionary distance [14]; in the second kind, regularities identified in genetic sequences by compression algorithms are used to represent biological significance for evolutionary history, but these data compression based methods often involve of aggregated errors [15]; in the third category, the evolutionary distance is measured by string composition based on the singular value decomposition (SVD) of a string frequency matrix [13], or on the composition vector on short strings of a fixed length or the information discrepancy on short strings of a fixed length [1,2]. In this paper, we use the cosine distance introduced in [4,5] as the evolutionary distance between the objects.

Definition 2: The evolutionary distance between objects

The evolutionary distance *d*(*object*_*i*_, *object*_*j*_) between *object*_i _and *object*_j _is defined as:



Here, C (object_i_, object_j_) is the cosine of the angle between vector *i *and vector *j *defined in [3,4].

Definition 3: The mean distance and the shortest distance

We use *d*_*mean*_(*object*_*i*_) to denote the mean distance from *object*_i _to all the other objects, namely



We also denote the shortest distance from object_*i *_to all the other objects as *d*_min_(*object*_*i*_).



Definition 4: The acceptance weights

For two objects *object*_*i *_and *object*_*j *_the acceptance weights for *object*_*i *_to *object*_*j *_is defined as Eq.(8):



Similarly, the acceptance weights for *object*_*j *_to *object*_*i *_is as Eq.(9):



From the definition we can see that the more similar two objects are, the greater acceptance weight to each other will be. We also can see that acceptance weight between two objects is not symmetric, namely, normally

accept_j _(object_i_) ≠ accept_i_(object_j_)          (10)

According to the definitions above, we could form a weighed digraph where each vertex represents an object. Denote the weight of the directed edge from object_i _to object_j _as τ_*ij*_(0). This value will be updated according to the pheromone deposited by the ants passing it. Its initial value τ_*ij*_(0) is set as the acceptance weight:

τ_*ij*_(0) = *accept*_*i*_(*object*_*j*_)          (11)

In traditional ant colony algorithm, pheromone on all edges is usually initialized as zero. This is not helpful for ants to choose path at the early stages. However, in AAPTC, the proposed initial pheromone value set on the digraph is much important for ants' latter movements, that is to say it can make great influence on the initial topology of the phylogenetic trees. Based on this initial value, in the latter stages the ants will update this pheromone digraph for the construction and optimization of the phylogenetic trees.

### Heuristic function

The heuristic function ηij in Eq.(1) is a problem dependent function that measures the "quality" of the edge (*i, j*) which connects the vertexes i and j representing the two objects. Here the "quality" means the preference of the edge to be selected by the ants. Obviously, the less distance between the two connected objects, the more preferred the edge should have. Therefore, η_ij _should be associated with the distance between objects. So it is given by the following formula.

η_*ij *_= 1/*d *(*object*_*i*_,*object*_*j*_)     (12)

Different from pheromone τ_ij_, η_ij _is static and unidirectional heuristic information determined by the distance information.

Pheromone Updating

In the algorithm, based on the following formula, pheromone on edge (*i*,*j*) is updated on the paths the ants just passed after each iteration.



Here constant ρ ∈ (0,1) is the coefficient of evaporation. At an individual iteration the pheromone on each path will be evaporated by a rate of ρ.

In the local updating period,  is the increment of τ_ij _by ant k, which is defined by Eq.(14)



Here Q is a constant. From the formulas above, it is easy to see that the more ants pass through an edge, the more pheromone deposited on it, and the more probability for the two vertexes connected by the edge to be included in the same strong connected component of the weighted digraph constructed in the third stage of the algorithm. In the global updating period,  is the increment of *τ*_*ij *_by sub-colony *k*, which is defined as follows :



Here, Tree_k _denotes the phylogenetic tree constructed by sub-colony_k_, fitness(sub-colony_k_) is the fitness value of Tree_k_. According to [9], once the topology and the branch lengths of Treek are determined, a new distance matrix can be deduced. By comparing this distance matrix D^*k *^with the original distance matrix D (calculated from the given objects), a quality measurement showed in Eq.(16) can be assigned to the tree as its fitness value:



The summation extends over all n(n-1)/2 distances between the n objects. If the distances are concentrated within a narrow scope, a high fitness value will be assigned to the tree, and if the reconstructed distance equal the original distances, the fitness will reach the highest value C. By global pheromone updating, the pheromone deposited on the edges of high fitness valued trees will be much higher than others, thus the objects connected by these edges can hardly be separated by the ants during the clustering process.

### Updating Parameters

The second stage of the algorithm consists the step of updating the value of α, β which are the parameters of the Eq.(1) which is the probability distribution for the ant's selecting the next vertex. In Eq.(1), parameters α, β determine the relative influence of the trail strength *τ*_*ij *_and the heuristic information *η*_*ij*_. At the initial stage of the algorithm, the pheromone value on each edge is relatively small. To speedup the convergence, the ants should select the path mainly according to the heuristic information *η*_*ij*_. Therefore, the value of α should be relatively large in this stage. After some iteration, the pheromone values on the edges are increased, their influence become more and more important. Therefore the value of β should be relatively large. Since the adjustment of the values of α and β should be based on the strength of pheromone on the edges In Eq.(17) we define τ_ave _as the average amount of pheromone on the pheromone digraph and in Eq.(18) define δ as the pheromone distributing weight to measure the distribution of pheromone on the graph.





Using the pheromone distributing weight δ, the algorithm updates the value of α, β as follows:

α = log ^1+*δ *^    (19)



Once the pheromone digraph is updated, α and β will be adaptively modified by the pheromone distributing weight and make influence on the effect of pheromone and heuristic function. By adjusting the value of α, β adaptively, the algorithm can accelerate the convergence and also can avoid local convergence and precocity. Therefore, this adaptive procedure is much important for AAPTC. Furthermore, since the amount of pheromone is an important measure for tree construction, the pheromone distributing weight δ is also a critical factor to terminate the iterations of the algorithm.

## Authors' contributions

LQ conceived, designed and performed the study under the supervision of LC and YP; LQ and YC (Yixin Chen) collected and analyzed the data; LQ wrote the computer code; LQ and YC designed algorithms; LQ and LC wrote the manuscript; All authors have read and approved the final manuscript.

## Figures and Tables

**Table 1:** The experimental results on the first data set.

Dataset	GA				AAPTC			
	
	mean	S.D.	high	low	mean	S.D.	high	low
S11	8.78	0.06	9.81	7. 40	9.83	0.02	9.94	9.77
			(68345)	(42157)			(65487)	(382914)
S12	5.39	0.35	9.75	5.11	9.67	0.04	9. 81	9.62
			(59623)	(34728)			(57631)	(26738)
S13	9.64	0.11	9.83	8.92	9.88	0.06	9.96	9.85
			(72100)	(32572)			(76895)	(15201)
S14	5.78	0.42	9.69	5.03	9.86	0.07	9.91	9.70
			(69805)	(25437)			(82965)	(43346)

Table 1 gives the results on the first set of simulated data. The optimal tree topology of these data is known in advance because they are generated for a predefined tree topology, and the fitness value of the optimal solution is just 10. Form experimental results given in table 1, both methods could find the optimal tree topology. In the cases of S11, S12, S13 and S14, AAPTC finds the optimal topology for all trials, whereas the GA method falls into local convergence eight times with average fitness value of 5.39 and six times with average fitness value of 5.78 on S12 and S14 respectively. Therefore, ACTP can get more global and higher fitness valued phylogenetic trees than GA.

**Table 2:** The experimental results on the second data set.

Dataset	GA				AAPTC			
	
	mean	S.D.	high	low	mean	S.D.	high	low
S21	9.12	0.06	9.79	9.03	9.67	0.01	9.88	9.63
			(53135)	(42257)			(78753)	(47030)
S22	9.04	0.11	9.22	8.98	9.81	0.03	9.92	9.73
			(69546)	(32419)			(68964)	(56229)
S23	8.87	0.17	9.14	8.25	9.87	0.05	9.89	9.83
			(57453)	(34565)			(76843)	(36457)
S24	8.90	0.21	8.91	8.56	9.90	0.09	9.98	9.84
			(56739)	(38897)			(66753)	(49332)

Table 2 shows the results on the second set of simulated data. We can also see that GA is more likely to fall into local convergence when the objects increase. But AAPTC can get the optimum from given data sets with large number of objects. And AAPTC proposes a more powerful phylogenetic clustering method so it can obtain high qualified solutions no matter how large the number of the objects extends.

**Table 3:** The average iterations.

Dataset	GA			AAPTC		
	
	mean	high	low	mean	high	low
S11	0.25	0.41	0.19	0.19	0.17	0.28
S12	0.38	0.43	0.32	0.26	0.39	0.22
S13	0.36	0.48	0.30	0.34	0.45	0.31
S14	0.55	0.62	0.46	0.42	0.47	0.39
S21	0.26	0.35	0.22	0.15	0.13	0.20
S22	0.34	0.44	0.28	0.29	0.37	0.24
S23	0.48	0.58	0.43	0.44	0.38	0.55
S24	0.62	0.68	0.56	0.51	0.46	0.59

Table 3 lists the computation time (hours) these two methods required to get the optimal solution from given data sets. From the experimental results on S11, S12, S21 and S22 we can see that, at the same condition, AAPTC saves much more time than GA.

**Table 4:** The comparison for fitness value between GA and AAPTC.

Dataset	GA				AAPTC			
	
	mean	S.D.	high	low	mean	S.D.	high	low
GPCR's	8.92	0.12	9.13	8.76	9.80	0.04	9.91	9.65
			(51535)	(41272)			(87689)	(38264)

It is clear from Table 4 that in case of the real data set, all methods give approximately the same results. Comparison between AAPTC and GA reveals that the AAPTC finds slightly better phylogenetic trees, according to the fitness values.
